# Supplementary material for: Nutritional analysis of commercially available, complete plant- and meat-based dry dog foods in the UK
Source: PLoS One. 2025 Sep 3;20(9):e0328506. doi: 10.1371/journal.pone.0328506 (PMC12407485; doi:10.1371/journal.pone.0328506)
Supplement: Attachment — Submitted filename: Rebuttal to revews 13-05-2025.docx [file pone.0328506.s003.docx]

Reviewer Comments:

**Reviewer #1**

1. Respectfully should read respectively line 7, para1 page 14

Changed. Page 7 line 7

2. Page 22 para 1 line 15 Reword the following to remove word churlish “It is almost churlish to propose, therefore, that for all foods designated as ‘complete,’ an assigned nutritional minimum for all essential and conditionally-essential amino acids should at least be followed”

Removed. Page 15 line 15

3. I think this reference should be dated 2021 not 2022. Also, these Guidelines have now been updated in 2024.

Updated reference to current (2024). Page 8 line 21

**Reviewer #2**

Thank you for your interesting study, and helping to advance this field. I’ve recommended Minor revisions.

Thank you to the reviewer for your kind words, in an often unkind arena. We have responded to your comments in turn below.

Comment: supplementation of plant-based foods.

We note your points and have modified the text accordingly, in places (see marked-up red). I do not think it would come as any surprise that vegetarian/vegan pet foods, in line with plant-based human dietary patterns, would be low in some B-vits and iodine. These were consistent in all our, albeit small, sample of plant-based foods. Thus, we suggest that certain supplementation could be considered, without side effects. For the other diets, then isolated – but not consistent – deficiencies were noted (e.g. some essential amino acids in renal foods) and we have noted the overall lack of compliance throughout the manuscript, including the discussion. Indeed, we do agree with your point and have modified the text accordingly.

***To do: It should be more prominently noted for context (where deficiencies or supplementation are described, including the Abstract), that varying nutritional deficiencies and imbalances are common across most diet types (including non-plant based, ie meat-based diets), and indeed that in this study no foods were found to be without flaws (e.g. “No food met FEDIAF guidelines for all nutrients.”***

Para 1 citation should be corrected to Vegan Society

Changed Page 3, line 5

Add citation to end of 1st sentence

added

References

It is how endnote formats non-standard references. Will try to alter, but can do after the fact by hand.

Tables

Readability hopefully improved.

Table 2

Yes, values for iron and magnesium as mgs per 1000kcal according to FEDIAF 2024 are indeed 9.00 and 0.18 (mgs per 1000 kcal ME), respectively. Page 16 of 98 in FEDIAF 2024.

Table 2 notes:

Yes, have altered to improve readability

Table 3

Added ‘B’ to make clearer
