# Supplementary material for: Nutritional analysis of commercially available, complete plant- and meat-based dry dog foods in the UK
Source: PLoS One. 2025 Sep 3;20(9):e0328506. doi: 10.1371/journal.pone.0328506 (PMC12407485; doi:10.1371/journal.pone.0328506)
Supplement: Attachment — Submitted filename: Rebuttal to revews 06-06-2025.docx [file pone.0328506.s004.docx]

Reviewer Comments:

**Reviewer #1**

No further corrections

**Reviewer #2**

My only remaining suggestion is to review boldfacing applied in tables where p < 0.05. A very few instances where p < 0.05 seem to have been missed

Tables with P = <0.05 have, I hope, now all been bold-faced.
